# A supply chain perspective on India’s COVID-19 crisis: Lessons from the second wave and preparing for a potential third wave

**DOI:** 10.7189/jogh.11.03116

**Published:** 2021-10-23

**Authors:** Karthik V Natarajan, Shailendra Prasad

**Affiliations:** 1 Supply Chain and Operations Department, Carlson School of Management, University of Minnesota, Minneapolis, Minnesota, USA; 2 Center for Global Health and Social Responsibility, University of Minnesota, Minneapolis, Minnesota, USA

April 2021 saw a significant upsurge in new COVID19 cases in India in what is now referred to as a ‘second wave’ [1]. The public health response was hindered by a lack of clarity and coordination between the national and state governments regarding roles and responsibilities, and a general state of chaos [2]. The highly fragmented nature of the Indian health care system also made it difficult for people to identify the right channels to access critical medical supplies. While different factors (eg, lack of sufficient ICU beds and oxygen manufacturing capacity) have been blamed for the breakdown of the health system, a siloed analysis does not get to the root causes behind the system failure.

We contend that an *end-to-end supply chain view* of the health system is required to identify the key factors that hampered the public health response. Our analysis of the supply chain challenges witnessed during the second wave in India (and COVID19 waves in other countries) reveals three important attributes that contributed to the health system breakdown – limited supply chain visibility, lack of coordination of sourcing and resource allocation, and limited buffer capacity to quickly ramp up supply. Accordingly, we develop three independent, but connected, building blocks foundational to building *resilient and responsive health care supply chains* that are critical to delivering effective responses in case of future COVID19 waves. We take a cross-cutting perspective by combining key principles from *supply chain* and *health care management* to outline strategies to narrow the supply-demand divide, while considering important factors critical to successful implementation.

## DEVELOPING RESILIENT AND RESPONSIVE SUPPLY CHAINS

### Building block 1 – Information visibility and cohesive data architecture

Companies have been investing heavily in *supply chain information systems* such as point-of-sales software (to collect consumer demand data) and blockchain technologies (to gain visibility into upstream product movement) to expeditiously fulfil customer demands [3]. While demand forecasting and information visibility play a vital role across industries, they assume heightened significance in health care supply chains since providing care often requires a “bundle” of essential supplies including hospital beds, therapeutics, and PPEs. Even if one item in the “bundle” is not available, it could lead to care interruption or delays. Ensuring consistent and reliable data availability from different nodes of the health care system and making that data “visible” to the relevant stakeholders are both vital to creating a robust resource allocation framework that can dynamically respond to rapidly evolving needs during health disasters.

**Figure:**
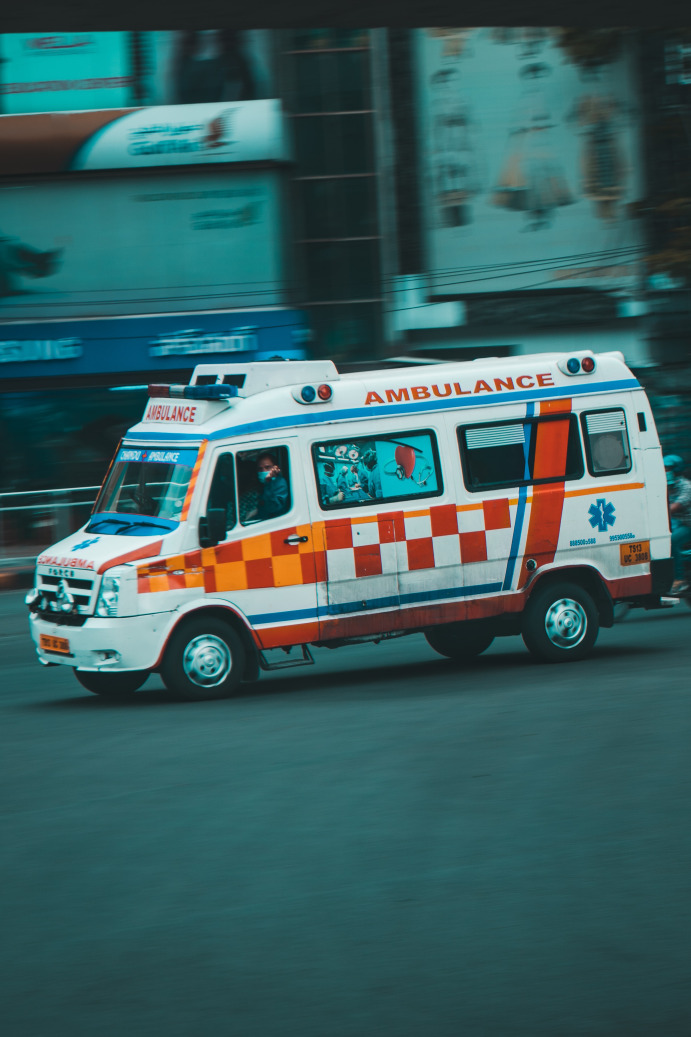
Photo: An ambulance rushing to the hospital in Hyderabad (https://unsplash.com/photos/T1C9zpFpky4).

During the pandemic, there has been a fragmented data approach across different Indian states. For example, states like Delhi provide the public with a high-level view of ICU beds availability [4]. while Tamilnadu provides a detailed breakdown including regular, ICU, and oxygen-supported beds [5]. Publicly disclosing hospital bed availability could alleviate supply-demand imbalances but more needs to be done to develop a coherent supply chain information strategy. Specifically, we call for the following to be included as part of such a strategy: (i) *systematic collection of test data* from all public and private COVID testing centers. This data, when combined with genomic sequencing, would help determine current needs and develop reliable forecasts of future demand on the health care system, (ii) *building an inventory database of critical medical supplies* within the public and private channels. Obtaining inventory information from private players on an ongoing basis will be challenging but targeted legislations requiring private firms to disclose such information during disasters would be instrumental in rapidly establishing supply chain information systems for effective pandemic response. Some entities within private channels already disclose inventory information (eg, hospital bed availability) and we advocate for expanding disclosure to cover other parts of the care “basket” (eg, drugs and oxygen cylinders) so that policymakers can better evaluate the health care system’s ability to make available the entire “basket” of supplies necessary to deliver care.

### Building block 2 – Coordinated sourcing and resource allocation

*Supply chain management* is the management of all activities involved in ensuring that the right product reaches the customer at the right place and at the right time [6]. Executing this becomes especially challenging during disasters since the needs at the different system levels (eg, state, district, and hospital/clinic) evolve dynamically over time. Furthermore, in case of multi-location events like pandemics, the peak caseload may be reached at different times in the different regions. During disasters, a “panic” mindset often sets in, and entities place larger orders than what might be required due to fear of shortages. Such order inflation distorts the true demand and results in rationing that may not be reflective of the immediate needs in each region. These factors highlight the need to set-up a supply chain command center, that, when aided by a cohesive data architecture as described above, can mitigate this problem by allocating supplies commensurate to the regions’ needs while also helping to coordinate and dynamically shift resources from ‘non peak-demand’ regions to ones experiencing peak demands.

A supply chain command center could also help to leverage economies of scale in sourcing. Without a coordinated sourcing effort, individual entities including hospitals, cities, and states are left to compete to source critical supplies. In the initial months of the pandemic in the US, there was no central entity managing the pandemic response supply chain. Instead, the response operations were largely left to the individual states, resulting in an intense bidding war where states competed against one another and the federal government to buy scarce medical supplies [7]. We have witnessed a similar non-coordinated response to the second COVID19 wave in India, with responsibility passed on to individual states. The dire need has been met on an ad hoc basis through a combination of supply chains involving government and private entities and donations, with no clearly identified channels to obtain needed supplies. In addition, the ad hoc approach has also resulted in medical supplies being sold on the black market at astronomical prices. A supply chain command center that coordinates the sourcing, resource allocation, and logistics operations could help to avoid the above-described problems. However, a fully centralized approach is not likely to be successful in India and other countries with diverse population groups and where health is considered the responsibility of states or local governing bodies. Instead, a blend of centralized and decentralized strategies, as discussed below, is likely to be more successful in tackling the future waves of COVID-19.

### Building block 3 – Supply chain mapping and risk assessment

*Supply chain mapping* refers to documenting information across companies involved in manufacturing a product, to create a global map of the supply network [8]. In light of the shortages of critical medical supplies witnessed during the second wave in India, there is an immediate need to conduct supply chain mapping to identify the sources of supplies that are part of the “care basket” and the risk associated with those sources [9]. If the mapping reveals that a particular commodity is “risky” due to reliance on one supplier or if scaling capacity is not possible in the event of a disaster, then the government needs to take proactive steps to develop alternate sources of supply or create stockpiles of specific items.

For example, the US faced shortages of PPEs and ventilators at the start of the pandemic during April-June 2020. Low domestic manufacturing capacity and extensive reliance on a few countries for sourcing made it difficult to ramp up supply to meet the emergent demand. Following a US government ordered review of the supply chains, efforts are now under way, with financial incentives and legislations, to bring manufacturing of some active pharmaceutical ingredients (APIs) back to the US and boost domestic manufacturing capacity of protective equipment. It is imperative that India also undertake supply chain mapping for critical equipment and therapeutics that were in short supply so that the country is better equipped to handle future waves of COVID19.

## CONCLUSIONS AND IMPLEMENTATION CONSIDERATIONS

To ensure the building blocks of a resilient supply chain are effective, decision makers must be cognizant of the operating environment in which such a supply chain would operate. One such factor is the governance structure where comparisons with India’s neighbor, China, are natural since the two countries have similar population sizes. China’s centralized political structure is ideally suited for a coordinated supply chain response to an epidemic. However, with the federal structure of India, and with individual states in charge of health, an adaptive supply chain strategy that blends centralized and decentralized approaches is required [10] – for example, procurement of critical supplies, coordinating aid flowing into the country, and developing strategies to ramp up domestic production should be under the purview of central agencies; state and local governing bodies are best positioned to perform allocation and distribution at the local level since they have a better understanding of the community needs.

When considering an adaptive supply chain strategy as described above, it is important to be mindful of the bureaucratic challenges that might arise during implementation. Experience from the second COVID19 wave in India suggests that the responsibility for payment (central vs state governments) for the centrally procured supplies is likely to be a contentious issue. Determining fair and equitable allocation policies to distribute the procured supplies to different states is also likely to be a challenge. Given that addressing these issues will require careful deliberation and negotiation between states and the central government, we recommend taking a proactive approach to promptly start working towards an overarching framework that will guide procurement and allocation decisions during future health emergencies.

There is an urgent need to build *resilient and responsive supply chains* that will be critical to tackling future waves in India and other countries. We believe that the principles laid out in this article serve as a useful first step in that direction.
